# A simple transmission dynamics model for predicting the evolution of COVID-19 under control measures in China

**DOI:** 10.1017/S0950268821000339

**Published:** 2021-02-10

**Authors:** Chenjing Shang, Yang Yang, Gui-Ying Chen, Xiao-Dong Shang

**Affiliations:** 1 Shenzhen Key Laboratory of Marine Bioresource and Eco-environmental Science, College of Life Science and Oceanography, Shenzhen University, Shenzhen 518060, China; 2 Institute of Deep-Sea Science and Engineering, Chinese Academy of Sciences, Sanya 572000, China; 3 State Key Laboratory of Tropical Oceanography, South China Sea Institute of Oceanology, Chinese Academy of Sciences, Guangzhou 510301, China; 4 Southern Marine Science and Engineering Guangdong Laboratory, Guangzhou 511458, China

**Keywords:** Coronavirus, COVID-19, growth rate, prediction model

## Abstract

Epidemic forecasting provides an opportunity to predict geographic disease spread and counts when an outbreak occurs and plays a key role in preventing or controlling their adverse impact. However, conventional prediction models based on complex mathematical modelling rely on the estimation of model parameters, which yields unreliable and unsustainable results. Herein, we proposed a simple model for predicting the epidemic transmission dynamics based on nonlinear regression of the epidemic growth rate and iterative methods, which is applicable to the progression of the COVID-19 outbreak under the strict control measures of the Chinese government. Our model yields reliable and accurate results as confirmed by the available data: we predicted that the total number of infections in mainland China would be 91 253, and the maximum number of beds required for hospitalised patients would be 62 794. We inferred that the inflection point (when the growth rate turns from positive to negative) of the epidemic across China would be mid-February, and the end of the epidemic would be in late March. This model is expected to contribute to resource allocation and planning in the health sector while providing a theoretical basis for governments to respond to future global health crises or epidemics.

## Introduction

In December 2019, the outbreak of COVID-19 that could cause severe respiratory symptoms and even deaths emerged in Wuhan, Hubei province, China [1, 2]. This new coronavirus was confirmed to be able to transmit between humans on 20 January 2020, significantly increased the risk of international spread [3, 4]. To mitigate the spreading of the epidemic, the Chinese central government progressively implemented the highest-level metropolitan-wide quarantine control in Wuhan city and 31 provinces since 23–24 January 2020. In the meantime, scientists are racing to characterise the virus, model epidemics and develop diagnostic reagents and vaccines [4–8].

As of 26 February, the spreading of COVID-19 has been reported in 52 countries, and the number of confirmed cases worldwide has reached 83 389. The progression of the epidemic has aroused widespread concerns of scientists. The epidemiological community has long used the basic reproduction number *R*_0_ to describe the spread of epidemics. It can be thought of the expected number of cases directly generated by one case where all individuals are susceptible to infections in the case of natural transmission of the virus. At present, many groups have estimated the *R*_0_ value of COVID-19 (1.4–6.47, average 2–3) through different models, but the results differ greatly and are far from reality [8, 9]. On 16 January, Neil Ferguson's team at Imperial College predicted that there would be 1723 infections in Wuhan on 12 January, and total infections worldwide would reach 100 000 by 26 January [10]. Wu predicts the total infections in Wuhan will reach 75 815 on 25 January [9]. Huang and Qiao proposed a data-driven model to predict the peak of the outbreak [11]. As the Chinese government has implemented stringent highest-level health intervention, none of these models are suitable for predicting the development of epidemics under this circumstance. Finding an accurate and simple predictive dynamic model is the key for predicting the evolution of the epidemic.

Herein, we proposed a simple model based on non-linear regression and iterative methods to predict the progression of the epidemic under stringent governmental control. Using this model, we predicted the inflection point and end time of the epidemic, the expected number of infected patients and the maximum number of beds required for regions including Wuhan, Hubei province, Guangdong province and mainland China. This model can help the government to prepare in advance in the allocation of medical resources and the deployment of medical staff in the event of an epidemic crisis.

## Materials and methods

We selected four representative datasets for our analysis: Wuhan, Hubei province (excluding Wuhan), Guangdong province and mainland China (excluding Hubei). Hubei province (excluding Wuhan), which surrounds Wuhan city, was selected for the study as it is the centre of COVID-19 outbreak, Wuhan. Guangdong province was chosen as it has the largest number of confirmed cases after Hubei province, with two large floating population and economically developed cities Guangzhou and Shenzhen. Mainland China (excluding Hubei) reflects the overall development of epidemics across the country. The data were from the official website of the National Health Commission of the People's Republic of China from 15 January to 18 February 2020.

Detailed descriptions of the method are covered in the rest of the paper.

## Results and discussion

The methods we use are the non-linear regression and iterative methods commonly used in the study of natural science [12, 13]. Compared with the Susceptible-infected-removed (SIR) and susceptible-exposed-infectious-recovered (SEIR) models used in epidemiology, our method is simple, accurate and reliable.

The daily growth rate of confirmed cases is calculated as follows:
1
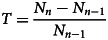

where *N*_*n*_ and *N*_*n*−1_ are the number of confirmed cases on day *n* and *n* − 1, respectively.

**Fig. 1.**
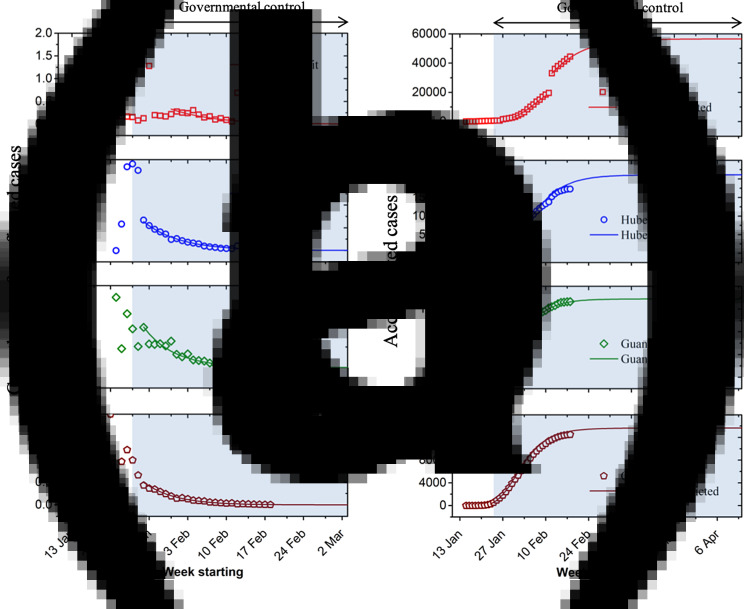
(a) Growth rate of confirmed cases and (b) accumulated cases for Wuhan, Hubei province, Guangdong province and mainland China. Asterisks denote Wuhan or Hubei is excluded (*: Wuhan excluded; **: mainland China excluded Hubei province). Red square for Wuhan, blue circle for Hubei, green diamond for Guangzhou and brown pentagon for mainland China.

The daily growth rate of confirmed cases is obtained by using formula (1) and is plotted in Figure 1a, where the hollow markers are calculated from real data and the solid lines are the fitted curve. The growth rate *T* fluctuates during the early stage of the outbreak and shows a large value because of a lack of governmental health intervention as well as the small sample size has a greater impact on *T*. *T* stabilises and decreases rapidly after 23–24 January 2020 due to nationwide quarantine policy. This growth rate curve decays exponentially and is obtained by fitting:
2
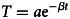

Here, *a* is a constant that represents the growth rate at *t* = 0, *β* is an attenuation coefficient that indicates the efficiency of government isolation and quarantine and *t* is the time representing the evolution of the epidemic. Formula (2) shows the evolution of the epidemic under the government's stringent isolation and quarantine of patients. The attenuation coefficient *β* is a parameter that measures the efficiency of isolation and quarantine.

Note that the Chinese central government implemented nationwide quarantine policy from 23–24 January 2020. Since there were delays for the control measures to take effect, except for Wuhan, we used the data starting from January 26 for model fitting (Fig. 1a). On the contrary, due to the shortage of monitoring tools and medical resources, the data of Wuhan during the early stage cannot truly reflect its real epidemic situation. We therefore used data of Wuhan starting from 31 January for model fitting. Furthermore, the jump in confirmed cases reported in Wuhan on 12 February is due to a change in the criteria for counting diagnoses of the virus.

The summary of fitted parameters is displayed in Table 1. The trends of the four curves are comparable (Fig. 1), which indicates that under the mandatory quarantine by the Chinese government, the transmission and evolution of the epidemic are artificially limited. The values of fitted attenuation coefficient *β* of the four curves show an increasing trend (Wuhan: 0.13; Hubei: 0.15; Guangdong: 0.18 mainland China: 0.18). We speculate that the faster the growth rate decays, the greater the government's impact over the epidemic. Note that the outbreak in Wuhan was not well controlled at the early phase of the outbreak, which led to the rapid spread of the outbreak and the rapid increase in the number of infected patients. Therefore, the attenuation coefficient of the growth rate is the smallest. The attenuation coefficient for mainland China (excluding Hubei province) is similar to that of Guangdong province, indicating that the extent of public health intervention is comparable. The smaller attenuation coefficient of Hubei province compared to the rest of China is possibly due to the challenge of implementing the control measures because of the overwhelmed medical system in Hubei.

**Table 1.** List of fitted parameters for formula (2)

Parameter	Wuhan	Hubei^a^ province	Guangdong province	China^b^
*a*	2.503	3.420	3.657	3.553
*β*	0.126	0.153	0.182	0.179
*R*^2^	0.905	0.981	0.929	0.984

a Wuhan excluded.

b Mainland China excluded Hubei province.

Based on formula (2), we use nonlinear regression and iterative methods to predict cumulative confirmed cases:
3
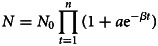

where *N* and *N*_0_ are the number of confirmed cases and the expected number of cases at *t* = 0, respectively.

Using formula (3), the cumulative numbers of confirmed cases over time for Wuhan, Hubei (excluding Wuhan), Guangdong province and mainland China (excluding Hubei) are predicted. Figure 1b shows the up-to-date confirmed cases (hollow markers) and predictions (solid lines) using our model for the four datasets. The model predictions are in good agreement with the actual numbers. Based on our model, the predicted total infections in Wuhan, Hubei (excluding Wuhan), Guangdong province and mainland China (excluding Hubei) during this outbreak are 56 519, 21 093, 1377 and 13 641, respectively (Table 3). The death toll can also be predicted in the same way. Furthermore, we used this model to predict the further development and evolution of the epidemic. Although the number of confirmed cases is increasing (Fig. 1b), the daily growth rate of confirmed cases is decreasing (Fig. 1a). It indicates that the move of the epidemic goes better. When the confirmed cases reach the peak, the epidemic tends to the end. Using this method, the estimated end time of the epidemic in Wuhan, Hubei (excluding Wuhan), Guangdong province and mainland China (excluding Hubei) will be 5 May, 13 April, 15 March and 27 March 2020, respectively. The mean absolute percentage error (MAPE) method was applied to evaluate the proposed model's prediction accuracy. The results are summarised in Table 2. An excellent short-term (between 02/17/2020 and 03/19/2020) prediction accuracy with an MAPE of less than 10% was obtained for all four subjects, except for Hubei province. Between 03/20/2020 and 04/16/2020, an increased MAPE was seen for all four subjects. The predictions for mainland China and Guangdong province remain excellent with an MAPE of less than 10%. The predictions for Wuhan and Hubei province are both between 10% and 20%.

**Table 2.** Prediction accuracy of the proposed model

Date	*E*_MAPE_			
	Wuhan (%)	Hubei^a^ (%)	Guangdong (%)	China^b^ (%)
02/17/2020–03/19/2020	8	16	1	3
03/20/2020–04/16/2020	13	19	8	8

a Wuhan excluded.

b Mainland China excluded Hubei province.

**Fig. 2.**
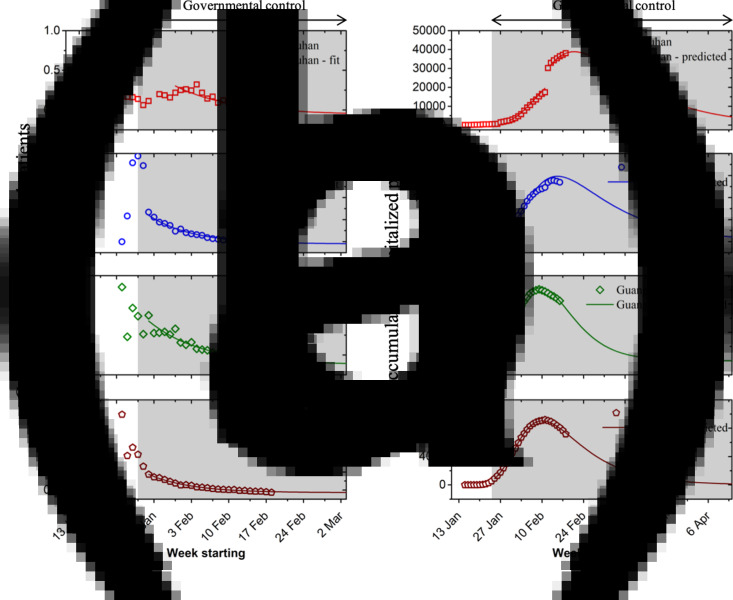
(a) Daily growth rate and (b) accumulated hospitalised patients for Wuhan, Hubei province, Guangdong province and mainland China. Asterisks denote Wuhan or Hubei is excluded (*: Wuhan excluded; **: mainland China excluded Hubei province).

Forecasting the number of hospitalised patients is conducive to the accurate preparation of beds and other resource allocation for the hospital, and provides significant guidance in response to the outbreak. The number of hospitalised patients, or the number of required hospital beds, can be obtained by subtracting the number of confirmed cases by the number of recoveries and deaths as follows:
4
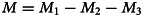

where *M*, *M*_1_, *M*_2_ and *M*_3_ are the number of hospitalised patients, confirmed patients, recoveries and deaths, respectively. Substituting formula (1) we obtain the growth rate of hospitalised patients for these four regions and the results are plotted in Figure 2a. The choices of symbols and colours correspond to those in Figure 1. This growth rate curve also follows an exponentially decaying trend and is obtained by fitting:
5
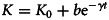

where *K* represents the growth rate of hospitalised patients, the sum of *K*_0_ and *b* represents the growth rate at *t* = 0 and *γ* represents the attenuation coefficient of the growth rate. The details of fitted parameters for formula (5) are provided in Table 3.

**Table 3.** List of fitted parameters for formula (5)

Parameter	Wuhan	Hubei^a^ province	Guangdong province	China^b^
*K*_0_	−0.050	−0.051	−0.120	−0.071
*B*	1.811	2.520	1.658	2.024
*Γ*	0.096	1.231	0.101	0.126
*R*^2^	0.838	0.973	0.914	0.989

a Wuhan excluded.

b Mainland China excluded Hubei province.

Then, we apply iterative methods to predict the number of hospitalised patients:
6
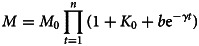

where *M*_0_ is the number of hospitalised patients at *t* = 0. The results are shown in solid lines in Figure 2b. Our predictions are in good agreement with existing data. The continuous increase in the number of hospitalised patients during the early phase (Fig. 2b) indicates that the number of new infections is greater than the sum of recoveries and deaths. At this stage, the development and spreading of the epidemic continues. When we reach the maximum value, the number of newly confirmed cases is equal to the sum of recoveries and deaths. When the number of patients in the hospital starts to decrease, it means that the number of newly confirmed patients is less than the sum of recoveries and deaths. When this curve reaches the maximum and begins to turn around, we call this point the inflection point. This point can also be seen in Figure 2a, that is, the point at which the growth rate changes from positive to negative. The peak value is the maximum number of beds required by hospitalised patients (Table 4). The maximum accumulated hospitalised patients for these four regions will be 38 888 (Wuhan), 14 849 (Hubei excluding Wuhan), 1029 (Guangdong) and 9057 (mainland China excluding Hubei). The outbreak in Wuhan overwhelmed the health systems. The patients have been forced to turn away due to a lack of beds and medical supplies. Accurately estimating the number of beds needed will assist the government in resource allocation and planning during a public health crisis.

**Table 4.** Summary of predictions using the proposed model

	Wuhan	Hubei^a^ province	Guangdong province	China^b^
Inflection point	2020/02/21	2020/02/15	2020/02/10	2020/02/10
Total required beds	38 888	14 849	1029	9057
Total cases	56 519	21 093	1377	13 641
End time	2020/5/5	2020/04/13	2020/03/15	2020/03/27

a Wuhan excluded.

b Mainland China Hubei province excluded.

Furthermore, our model's efficacy was compared with other studies using other models. For example, Dandekar *et al*. [14] showed an SIR model, when estimating the total cases by the end of February 2020 in Wuhan, China, can lead to an error of 150%. Wang *et al*. [15] employed an SEIR model, estimated 11 044, 70 258 and 227 989 confirmed cases by the end of February 2020 in Wuhan, China, using *R*_0_ = 1.9, 2.6 and 3.1, respectively. These estimations came down to an absolute error of 78%, 43% and 364%, respectively, far beyond our error of 8%. The authors stated that classical estimation models' accuracy largely depends on the parameters used, based on previous studies and various approaches and assumptions, and required a large dataset. Furthermore, the classical SEIR and SIR models' parameters are assumed to be constant. Therefore, they cannot recover the stagnation observed in Wuhan's infected case count due to the strict government control in China. Moreover, despite the numerous efforts to generate the model for Wuhan, the authors stated that the dynamics model for the other locations in mainland China and other places in the world still needs to be developed with specific parameters to be redefined.

## Conclusions

Several models for the prediction of the COVID-19 outbreak existed, but few could well predict the transmission of the epidemics under stringent public health intervention. Based on the growth rates *T* and *K*, we proposed a simple model for studying and predicting the progression of the epidemics. We evaluated the role of government control policies based on this model and predicted the inflection point and end time of the epidemic, the maximum number of hospitalised patients and the expected number of infections (Table 3). Our model yields accurate short-term forecasting of the pandemic's progression: our predictions are in excellent agreement with the real data. It should be noted that the proposed model will not be necessarily accurate for long-term predictions due to changes in the external environment (e.g. the update of the government control policies, development of the treatment methods, etc.). Therefore, model parameters will need to be timely updated to generate reliable long-term predictions.

In summary, we developed a simple yet effective model using a small dataset for predicting the evolution of the COVID-19 outbreak in mainland China. The mathematical model is of great guiding significance to assess the impact of government control policies in preventing the spread of the disease. This model is expected to contribute to resource allocation and planning in the health sector while providing a theoretical foundation for governments to respond to future global health crises or epidemics.

## Data Availability

The datasets generated during and/or analysed during the current study are available from the corresponding author on reasonable request.
